# Molecular circumscription and major evolutionary lineages of the fern genus *Dryopteris* (Dryopteridaceae)

**DOI:** 10.1186/1471-2148-12-180

**Published:** 2012-09-13

**Authors:** Li-Bing Zhang, Liang Zhang, Shi-Yong Dong, Emily B Sessa, Xin-Fen Gao, Atsushi Ebihara

**Affiliations:** 1 The ECORES Lab, Chengdu Institute of Biology, Chinese Academy of Sciences, P.O. Box 416, Chengdu, Sichuan, 610041, P. R China; 2 Missouri Botanical Garden, P.O. Box 299, St. Louis, Missouri, 63166-0299, USA; 3 South China Botanical Garden, Chinese Academy of Sciences, Guangzhou, 510650, P.R. China; 4 Botany Department, University of Wisconsin-Madison, 430 Lincoln Drive, Madison, Wisconsin, 53706-1313, USA; 5 Department of Botany, National Museum of Nature and Science, Tsukuba-shi, Ibaraki, 305-0005, Japan

## Abstract

**Background:**

The fern genus *Dryopteris* (Dryopteridaceae) is among the most common and species rich fern genera in temperate forests in the northern hemisphere containing 225–300 species worldwide. The circumscription of *Dryopteris* has been controversial and various related genera have, over the time, been included in and excluded from *Dryopteris*. The infrageneric phylogeny has largely remained unclear, and the placement of the majority of the supraspecific taxa of *Dryopteris* has never been tested using molecular data.

**Results:**

In this study, DNA sequences of four plastid loci (*rbcL* gene, *rps4-trnS* spacer, *trnL* intron, *trnL-F* spacer) were used to reconstruct the phylogeny of *Dryopteris*. A total of 122 accessions are sampled in our analysis and they represent 100 species of the expanded *Dryopteris* including *Acrophorus*, *Acrorumohra*, *Diacalpe*, *Dryopsis*, *Nothoperanema*, and *Peranema*. All four subgenera and 19 sections currently recognized in *Dryopteris* s.s. are included. One species each of *Arachniodes*, *Leptorumohra*, and *Lithostegia* of Dryopteridaceae are used as outgroups. Our study confirms the paraphyly of *Dryopteris* and provides the first strong molecular evidence on the monophyly of *Acrophorus*, *Diacalpe*, *Dryopsis*, *Nothoperanema*, and *Peranema*. However, all these monophyletic groups together with the paraphyletic *Acrorumohra* are suggested to be merged into *Dryopteris* based on both molecular and morphological evidence. Our analysis identified 13 well-supported monophyletic groups. Each of the 13 clades is additionally supported by morphological synapomophies and is inferred to represent a major evolutionary lineage in *Dryopteris*. In contrast, monophyly of the four subgenera and 15 out of 19 sections currently recognized in *Dryopteris* s.s is not supported by plastid data.

**Conclusions:**

The genera, *Acrophorus*, *Acrorumohra*, *Diacalpe*, *Dryopsis*, *Nothoperanema*, and *Peranema*, should all be merged into *Dryopteris*. Most species of these genera share a short rhizome and catadromic arrangement of frond segments, unlike the sister genus of *Dryopteris* s.l., *Arachniodes*, which has anadromic arrangement of frond segments. The non-monophyly of the 19 out of the 21 supraspecific taxa (sections, subgenera) in *Dryopteris* strongly suggests that the current taxonomy of this genus is in need of revision. The disagreement between the previous taxonomy and molecular results in *Dryopteris* may be due partly to interspecific hybridization and polyplodization. More morphological studies and molecular data, especially from the nuclear genome, are needed to thoroughly elucidate the evolutionary history of *Dryopteris*. The 13 well-supported clades identified based on our data represent 13 major evolutionary lineages in *Dryopteris* that are also supported by morphological synapomophies.

## Background

The fern genus *Dryopteris* Adans. (Dryopteridaceae) is estimated to contain 225 to 300 species worldwide [1,2]. The circumscription of *Dryopteris* has been controversial and various related genera have been included in and excluded from *Dryopteris* (see [1]). Christensen [3] divided *Dryopteris* into seven subgenera, six of which belong to today’s Thelypteridaceae. Van Alderwerelt van Rosenburgh [4] separated *Stenolepia* Alderw. from Malaysian *Dryopteris*. Christensen [5] also split *Stigmatopteris* C. Chr. from American *Dryopteris*. Later he [6] gave up his earlier treatment and recognized *Dryopteris* sect. *Stigmatopteris* (C. Chr.) C. Chr. Ching [7] separated *Lithostegia* Ching from Sino-Himalayan *Dryopteris*. Later, *Lastreopsis* Ching [8] and *Ctenitis* (C. Chr.) C. Chr. [9] were both separated from *Dryopteris*. *Nothoperanema* (Tagawa) Ching was established by Ching [10] based on *Dryopteris* subgen. *Nothoperanema* Tagawa [11]. Thinking “*Ctenitis* subgen. *Dryopsis* Ching” (nom. inval.; Art. 36.1, [12]) to be more closely related with *Dryopteris*, Holttum & Edwards [13] described *Dryopsis* Holttum & P. J. Edwards as a genus.

The phylogenetic positions of most of these genera have recently been clarified. *Stigmatopteris* and *Ctenitis* are both rather isolated within the dryopteroid lineage [14], while *Megalastrum* Holttum, a relatively recent segregate of *Ctenitis*, forms a clade with *Rumohra* Raddi and the paraphyletic genus *Lastreopsis*[14,15]. None of these genera are in fact closely related to *Dryopteris*.

Although the close affinity among some but not all of *Acrophorus* C. Presl, *Acrorumohra* (H. Itô) H. Itô, *Diacalpe* Blume, *DryopsisDryopterisNothoperanema*, and *Peranema* D. Don has long been noticed (e.g., [16,17]), it has been unclear exactly how they are phylogenetically related. In studying the historical biogeography of the species of Hawaiian *Dryopteris*, Geiger & Ranker [18] sampled 63 species of *Dryopteris* and found *Nothoperanema* (represented by one species) to be embedded within a paraphyletic *Dryopteris*. Using *rps4-trnS* sequence data of 60 Chinese species of *Dryopteris* and several related genera, Li & Lu [19] reinforced Geiger & Ranker’s [18] finding that *Nothoperanema* (three species sampled) should belong to *Dryopteris* and for the first time confirmed that *Acrorumohra* (one species sampled) belongs to *Dryopteris* as well. The inclusion of *AcrophorusDiacalpeDryopsis*, and *Peranema* in Dryopteridaceae was strongly supported by Li & Lu’s [20] and Liu et al.’s [21] works based on *rbcL* and *rbcL* + *atpB* data, respectively. With relatively small sampling, both of the works also found that *Dryopteris* is paraphyletic in relation to these genera plus *Acrorumohra*.

To date, Fraser-Jenkins [1] has carried out the most intensive taxonomic study on *Dryopteris* worldwide, partly on the basis of early such work carried out by Itô [22,23] on the Japanese species and by Ching [8] on species in China, the Himalaya, and neighboring areas. Fraser-Jenkins [1] recognized 225 species which he divided into four subgenera: *D.* subgen. *DryopterisD.* subgen. *Erythrovariae* (H. Itô) Fraser-Jenk., *D.* subgen. *Nephrocystis* (H. Itô) Fraser-Jenk., and *D.* subgen. *Pycnopteris* (T. Moore) Ching. He divided the former three subgenera further into 16 sections. In his series of studies of species of *Dryopteris* in Yunnan, China, Lu [2,24,25] proposed three new sections: *D.* sect. *Caespitosae* S. G. Lu, *D.* sect. *Chrysocomae* S. G. Lu, and *D.* sect. *Indusiatae* S. G. Lu, two of which were adopted by Wu & Lu [26] in their classification of the 127 Chinese species of *Dryopteris*. The non-monophyly of *D.* subgen. *Dryopteris* and *D.* subgen. *Pycnopteris* has been detected by Geiger and Ranker [18] and Li and Lu [19,20], respectively, using DNA sequences of one or two loci and with relatively small species-level sampling (ca. 60 species in both studies). Based on analyses of seven plastid loci and 97 *Dryopteris* species, Sessa et al. [27] rejected monophyly of eleven of Fraser-Jenkins’ [1] sections and three of the four subgenera. Several additional sections have never been tested for their monophyly using molecular data.

The goals of this study include: [1] vigorously testing the monophyly of *Dryopteris* by including representative species of every subgenus and every section of *Dryopteris* currently recognized and by including all controversially related genera; [2] resolving phylogenetic relationships between *Dryopteris* and *Acrophorus*, *Acrorumohra*, *Diacalpe*, *Dryopsis*, *Dryopteris*, *Nothoperanema*, and *Peranema*; [3] assessing the monophylies of supraspecific taxa at sectional and subgeneric ranks recognized in current classifications using relatively large sampling and DNA sequences of multiple loci; and [4] identifying major evolutionary lineages in *Dryopteris*.

## Results

### Analyses of individual plastid regions

The characteristics and statistics of four individual plastid regions from MP and ML analyses are presented in Table 1. The four individual plastid regions as well as the combined *trnL* intron and *trnL-F* spacer yielded similar tree topologies in both MP and ML analyses (trees not shown). The most parsimonious, parsimony JK, and likelihood JK and BS trees for all analyses are available upon request from the first author. There were no well-supported (≥70% JK or BS support; [28]) clades that conflicted with one another in both the parsimony JK and likelihood BS trees. Therefore, the four plastid regions were combined. 

**Table 1:** Data-matrix and tree statistics for each of the analyses

**Matrix**	**# Accessions**	**# Chars.**	**# PI chars.**	**% Miss. / inappl.**	**MPT length**	**# MPTs**	**# MP JK / ML JK(BS) clades**	**Avg. MP JK / ML JK(BS) support (%)**	**CI**	**RI**
*rbcL* gene	114	1,324	167	9	541	541	59 / 73	79 / 80	0.5360	0.8280
*rps4-trnS* spacer	92	1,166	184	26	534	534	46 / 58	81 / 83	0.6592	0.8409
*trnL* intron	68	700	149	45	376	376	39 / 51	87 / 85	0.6782	0.8775
*trnL-F* spacer	111	448	97	11	288	287	44 / 56	76 / 80	0.6111	0.8911
*trnL* intron &*trnL-F* spacer	111	1,148	248	11	674	674	60 / 74	82 / 84	0.6439	0.8817
simultaneous	125	3,638	599	0	1,831	1,785,800	93 / 96	81 / 85	0.5877	0.8370

“PI” = parsimony-informative. “% miss. / inappl.” = percentage of cells in the data matrix scored as missing or inapplicable. “MPT” = most parsimonious tree(s). “MP” = maximum parsimony. “ML” = maximum likelihood. “JK” = jackknife. “BS” = bootstrap. “CI” = consistency index. “RI” = retention index.

### Analyses of combined plastid data

The combined data matrix of four plastid regions consisted of 3,638 bases. A simultaneous analysis [29,30] of nucleotides from all plastid regions was conducted as the primary basis for phylogenetic inference in *Dryopteris*.

**Figure 1:**
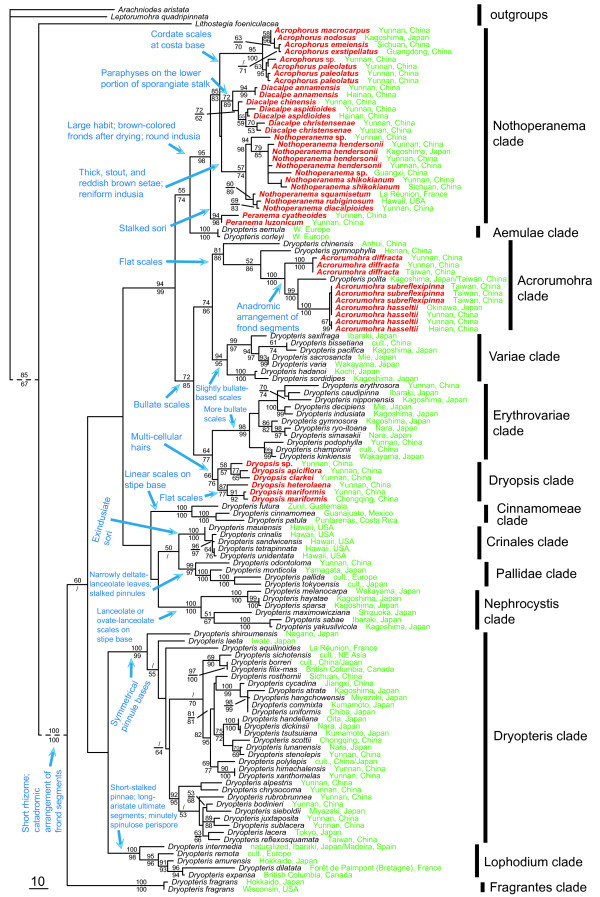
**Simultaneous-analysis maximum likelihood tree with parsimony jackknife values above each branch, and maximum likelihood bootstrap values below each branch.** If a clade was resolved in one analysis but not the other, “/” is used to indicate which analysis that clade was not resolved in. Dashed branches indicate the disproportional branch lengths. The species in red color indicate those that are currently not in the genus *Dryopteris* but resolved as members of *Dryopteris* in this study. Major morphological and/or palynological synapomorphies are indicated in blue. Geographical provenances are indicated in green.

Unweighted MP simultaneous analysis generated 1,785,800 most parsimonious trees with a length of 1,831 steps, a consistency index (CI; [31]) of 0.5877, and a retention index (RI; [32]) of 0.8370. The MP simultaneous analysis terminated prematurely when it was out of memory. The ML simultaneous analysis generated one optimal tree which is shown in Figure 1. The tree topology of the MP simultaneous analysis was similar to that from the ML simultaneous analysis and there existed no well-supported conflicts between the two trees. 

## Discussion

### The monophyly and circumscription of *Dryopteris*

Our analyses showed that *Dryopteris* in its current circumscription is paraphyletic in relation to *AcrophorusAcrorumohraDiacalpeDryopsisNothoperanema*, and *Peranema* (Figure 1). This resolution is consistent with that of Geiger & Ranker [18], in which only *Dryopteris* and one species of *Nothoperanema* were sampled. Our finding is also in accordance with that of Li & Lu [19] who sampled *AcrorumohraDryopteris*, and *Nothoperanema*. Sessa et al. [27] found *Dryopteris* to be monophyletic, but did not include representatives of any of these six genera in their sampling schemes. Our study provides the first strong molecular evidence that the traditionally defined Peranemataceae sensu Wu [33,34] and Wu & Ching [35] (*AcrophorusDiacalpe*, and *Peranema*), and *AcrorumohraDryopsis*, and *Nothoperanema*, should all be merged into *Dryopteris*, though each is monophyletic except *Acrorumohra*. The expanded *Dryopteris* is supported as monophyletic with strong support (Figure 1; ML BS: 100%; MP JK: 100%). Interestingly, except for *Acrorumohra* and *Nothoperanema*, and despite the similarity among members of these genera, the study of Li & Lu [20] was the first to suggest that *Dryopteris* was paraphyletic with respect to *Acrorumohra, Acrophorus, Diacalpe, Dryopsis* (included in *Ctenitis*), and *Peranamea*. Such a close relationship among all of them had not previously been suggested in the literature, although the close affinities among Peranemataceae, *Dryopsis* (previously in Tectariaceae), and Dryopteridaceae have partially long been noticed (e.g., [8,13,16,17,33]). Morphologically, Peranemataceae, *DryopsisDryopteris*, and *Nothoperanema* share short rhizome and catadromic arrangement of frond segments.

### Resolution of Peranemataceae

The family Peranemataceae was established by Ching [36]; as “Perenemaceae”] and is composed of *AcrophorusDiacalpe*, and *Peranema*[33-35,37,38]. Kramer [16] recognized *AcrophorusNothoperanemaPeranema* (including *Diacalpe*), and *Dryopsis* as independent genera, in addition to *Dryopteris* and another 24 or 25 genera (with one being Incertae Sedis), in his large subfamily Dryopteridoideae, one of the two subfamilies in Dryopteridaceae sensu lato (the other one is Athyrioideae). Recognition of these genera in Dryopteridaceae was largely followed by Smith et al.’s [17] classification. It is clear now that Dryopteridaceae sensu Kramer [16] are highly polyphyletic and contain the now separately circumscribed families Athyriaceae, Cystopteridaceae, Dryopteridaceae, Hemidictyaceae, Hypodematiaceae, Oncleaceae, Tectariaceae, and Woodsiaceae (e.g., [39,40]).

Morphologically, Peranemataceae can easily be distinguished from Dryopteridaceae by having slightly raised receptacles and inferior indusia that are coriaceous and globose or membranaceous and semi-globose [33-36]. However, the two families have the same basic chromosome number, x = 41. Morphologically, they also share catadromic arrangement of frond segments. The family Peranemataceae is generally not recognized in modern classifications (e.g. [16,17,39]). Our study shows that Peranemataceae sensu Ching [36], Wu [33,34], and Wu & Ching [35] are not monophyletic because *Nothoperanema*, normally not viewed as a member of Peranemataceae, is embedded within Peranemataceae. In our analyses, *Nothoperanema* and Peranemataceae together formed a strongly supported monophyletic group in our analyses (ML BS: 98%; MP JK: 95%), sister to the Aemulae clade within *Dryopteris* (see below). Our molecular data unambiguously resolved the species of Peranemataceae as members of *Dryopteris* (Figure 1).

### Resolution of *Acrophorus*

The genus *Acrophorus* has been recognized by numerous authors (e.g., [16,17,33-35,37-39,41,42]), but was not recognized by Fraser-Jenkins [43], who synonymized it with *Peranema*. *Acrophorus* is characterized by having a cordate and often persistent scale at the bases of costae, membranaceous and semi-globose indusia, a few multi-celled septate clavate paraphyses on the lower portion of the sporangiate stalk, and a few short multi-celled clavate appendages on the margins of scales at the stipe bases [33-35]. *Acrophorus* contains about 12 species occurring in Southeast Asia, westward reaching Papua New Guinea and Polynesia [44]. Six species are sampled in our study including the type, *A. nodosus* C. Presl. *Acrophorus* is strongly supported as monophyletic in our study (Figure 1; ML BS: 100%; MP JK: 95%). In the ML tree it is sister to *Diacalpe* + *Nothoperanema,* but this resolution received low statistical support. In the ML BS tree, it formed an unresolved trichotomy with *Diacalpe* and *Nothoperanema* (tree not shown). Based on our study, *Acrophorus* belongs to *Dryopteris*, and represents a specialized group within *Dryopteris* with round indusia and cordate scales at costa base.

Within *Acrophorus*, *A. paleolatus* Pic. Serm. is strongly supported as sister to the remaining species (Figure 1).

### Resolution of *Diacalpe*

*Diacalpe* is recognized by Pichi Sermolli [38], Wu [33,34], Wu & Ching [35], and Christenhusz et al. [39], but is synonymized with *Peranema* by Nayar & Kaur [45] who also included *Lithostegia* Ching in *Peranema*. *Lithostegia* has a close affinity with *Arachniodes* as shown by Liu et al. [21] and our unpublished data. Kuo [41], Kramer [16], and Smith et al. [17] also treated *Diacalpe* as a synonym of *Peranema*. Four species, including the type, *D. aspidioides* Blume, are sampled in our study. Our analyses show that *Diacalpe* is strongly supported as monophyletic (ML BS: 89%; MP JK: 72%) and *Diacalpe* and *Peranema* are paraphyletic in relation to *Acrophorus* and *Nothoperanema*, contrasting Nayar & Kaur’s [45], Kramer’s [16], and Smith et al.’s [17] treatments of *Diacalpe* as a synonym of *Peranema* while recognizing *Acrophorus*. Our resolution of *Diacalpe* is consistent with Liu et al. [21] where only two species of *Diacalpe* were sampled. Morphologically, *Diacalpe* is characterized by unstalked sori, a few single-celled long and clavate paraphyses on the lower portion of sporangiate stalk, and entire scales at the stipe bases [33-35].

Within *Diacalpe*, *D. annamensis* Tagawa is resolved as sister to the rest of species, with *D. chinensis* Ching & S. H. Wu then sister to *D. aspidioides* + *D. christensenae* Ching (Figure 1).

### Resolution of *Peranema*

With its two species occurring in tropical and subtropical Asia [46], the bitypic genus *Peranema* is recognized by nearly all pteridologists (e.g., [16,17,33-35,37-39,43,46]). *Peranema* is strongly supported as monophyletic in our study (Figure 1; ML BS: 98%; MP JK: 94%). This genus is morphologically distinguishable from *Diacalpe* by having stalked sori, no paraphyses on the lower portion of sporangiate stalks, and a few short and single-celled clavate hairs on the margins of scales at the stipe bases [33-35]. Synonymization of *Diacalpe* with *Peranema*, as done by Nayar & Kaur [45], Kramer [16], Smith et al. [17], and Fraser-Jenkins [43], is rejected by our data, which resolved *Peranema* as sister to a clade containing *AcrophorusDiacalpe*, and *Nothoperanema* (Figure 1). Although the morphological difference between *Dryopteris* and *Peranema* is striking, e.g., the presence of stalked sori in the latter, our data show that *Peranema*, like other members of Peranemataceae, should be merged into *Dryopteris*.

### Resolution of *Acrorumohra*

Originally described as a subgenus, *Rumohra* sect. *Acrorumohra* H. Itô [22], and later elevated to a genus [39], *Acrorumohra* is now widely recognized (e.g., [16,17,35,38,39,47]), though Fraser-Jenkins [1] subsumed it under *Dryopteris* sect. *Nephrocystis* (H. Itô) Fraser-Jenk. *Acrorumohra* is well defined morphologically. Its pinnules are all anadromous and the terminal pinnules have asymmetrical bases, different from *Dryopteris*. Ten accessions of three species of *Acrorumohra*, including the type, *A. diffracta* (Baker) H. Itô, are sampled in our study. Our analyses demonstrate, for the first time, that *Acrorumohra* is paraphyletic in relation to *Dryopteris polita* Rosenst. *D. polita* is sister to *A. hasseltii* (Blume) Ching plus *A. subreflexipinna* (Ogata) H. Itô, and together these three are sister to *A. diffracta.* Our results clearly show that *Acrorumorha* is a member of *Dryopteris*.

Recently, *Acrorumohra subreflexipinna* has been postulated to have arisen through recurrent hybridization between *A. hasseltii* and *A. diffracta,* with the former being its putative maternal parent and the latter its paternal progenitor [48]. Our resolution of these three taxa (Figure 1: Acrorumohra clade) supports *A. hasseltii* as the maternal progenitor of *A. subreflexipinna*.

### Resolution of *Nothoperanema*

Tagawa [11] originally described this taxon as a subgenus of *Dryopteris*. Ching [10] elevated it to a genus. *Nothoperanema* has been accepted at the generic level by many pteridologists (e.g., [35,37,38,41,47,49]). In contrast, Itô [50], Copeland [51], and Ohwi [52] regarded it to be part of *Ctenitis*. Smith et al. [17] and Christenhusz et al. [39] treated it as part of *Dryopteris* based on Geiger & Ranker’s [18] findings. The most important morphological synapomorphy of *Nothoperanema* is the presence of short and thick setae on each side of the costae and at the forking position of the midribs [10]. Eleven accessions of four species are included in our sampling, including the type of the genus, *N. squamisetum* (Hook.) Ching. For the first time, *Nothoperanema* is supported as monophyletic in our analyses (Figure 1; ML BS: 74%; MP JK: 57%), in contrast with Li & Lu’s [20] resolution where *Nothoperanema* was resolved as paraphyletic in relation to *AcrophorusDiacalpe*, and *Peranema*. Our study also confirmed Liu et al.’s [21] finding that *Nothoperanema* is embedded within a paraphyletic Peranemataceae. This resolution is accordant with the general morphological similarities except for differences in the morphology of the indusia between *Nothoperanema* and *Diacalpe*/*Peranema*[10]. Our study also reinforced Geiger & Ranker’s [16; with *N. rubiginosum* A. R. Sm. & Palmer only sampled] finding that *Nothoperanema* is nested within a paraphyletic *Dryopteris*, and we conclude that *Nothoperanema* should be a member of *Dryopteris*. The two share similar reniform indusia, short rhizomes, and catadromic arrangement of frond segments.

Within *Nothoperanema*, *N. diacalpioides* Ching, *N. rubiginosum*, and *N. squamisetum* together are strongly supported as sister to the remaining members of the genus. The relationship between *N. hendersonii* (Bedd.) Ching and *N. shikokianum* (Makino) Ching needs further clarifications.

### Resolution of *Dryopsis*

The genus *Dryopsis* was established by Holttum & Edwards [13] based on “*Ctenitis* subgen. *Dryopsis* Ching”. It is now widely recognized [17,39,53,54], though the relationships among *CtenitisDryopsis*, and *Dryopteris* have been controversial. Morphologically, *Dryopsis* appears to be more distant from *Ctenitis* than from *Dryopteris*. *Dryopsis* has distinct venation on the abaxial surfaces, sori terminal on veinlets, and marginal, entire scales that are clathrate or not, but with long and dull areolae. *Ctenitis* has venation indistinct on both the adaxial and abaxial surfaces, sori middle on the veinlets, and scales ciliate on their margins, clathrate, and with nearly hexagonal and lustrous areolae [8,13,54]. The major difference between *Dryopsis* and *Dryopteris* is that the former has either shallow or deep rachis and costa grooves that are closed near their bases, as well as multi-celled hairs with a thickened base on the margins but not in the grooves of the rachis and costae. *Dryopteris*, in contrast, always has deep rachis and costa grooves that are connected near their bases, and normally has no hairs on the rachis or costae [8,13,54].

*Dryopsis* contains about 22 species [55] occurring in tropical and subtropical Asia and reaching southwestward to southern India and Sri Lanka, eastward to Japan and the Philippines, and southward to Malaysia and Indonesia. It is most diverse in the southern and southeastern Himalaya [13,55]. With two species sampled, Liu et al. [21] discovered that *Dryopsis* should be a member of Dryopteridaceae but Liu et al. [21] failed to resolve the relationships among *DryopsisDryopteris*, and Peranemataceae sensu Ching [36,37], and Wu [33]. Liu et al. [21] also concluded that *Dryopsis* is not closely related to *Ctenitis*.

Six accessions of five species of *Dryopsis*, including the type*, D. apiciflora* (Wall. ex Mett.) Holttum & P. J. Edwards, are sampled in our analysis. Our results demonstrate that *Dryopsis* is monophyletic (ML BS: 76%; MP JK: 66%), in contrast to the resolution of Li & Lu [20], where three species of *Dryopsis* formed an unresolved trichotomy with two species of *Dryopteris* and one species of *Acrorumohra*. Our results also indicate that *Dryopsis* is nested within a paraphyletic *Dryopteris* (Figure 1), strongly suggesting that *Dryopsis* should be subsumed into *Dryopteris*. This resolution is not surprising given that the morphological difference between *Dryopsis* and *Dryopteris* is minute (see above).

Within *Dryopsis*, the species sampled were resolved into two clades. Morphologically, species of the upper clade (*D. apiciflora*, *D. clarkei* (Baker) Holttum & P.J. Edwards, and *D.* sp.) have bullate scales, while those of the lower clade (*D. heterolaena* (C. Chr.) Holttum & P.J. Edwards, *D. mariformis* (Rosenst.) Holttum & P.J. Edwards) have flat scales (Figure 1).

### Monophylies of supraspecific taxa in *Dryopteris*

Our 100-species sampling is still not dense enough to rigorously test the monophylies of all supraspecific taxa (sections or subgenera) recognized in recent classifications by Fraser-Jenkins [1], Lu [2,24,25], and Wu & Lu [26], given that *Dryopteris* s.s. contains between 225 [1] and 300 species [56], and in fact is even larger given that *DryopsisNothoperanema*, and Peranemataceae should be included in *Dryopteris* following our current work. However, our sampling allowed us to reject the monophylies of some supraspecific taxa because all four subgenera and 17 out of all 19 sections sampled (except *D.* sect. *Purpurascentes* and the monotypic *D.* sect. *Politae*) were represented by two or more species in our study (Appendix I).

The non-monophyly of the 19 out of the 21 supraspecific taxa in *Dryopteris* strongly suggests that the current taxonomy of this genus is in need of revision. However, our data do not necessarily falsify the monophyly of these 19 sections. The disagreement between previous taxonomy and molecular results in *Dryopteris* may be due partly to interspecific hybridization and polyplodization [57,58].

There are four well-documented allopolyploids in *Dryopteris* that have evolved via inter-clade hybridization, based on plastid *trnL-F* sequences, nuclear *PgiC* sequences, and/or biochemical evidence. *D. guanchica*, limited to Spain, Portugal, and the Canary Islands, has been postulated to be of hybrid origin between *D. aemula* (*D.* sect. *Aemulae*; our Aemulae clade) and possibly *D. intermedia* (*D.* sect. *Lophodium*) [57]. The Japanese endemic *D. shibipedis* Sa. Kurata, an obvious member of *D.* sect. *Variae* judging from the morphology [1,42], has possibly a hybrid origin between *D. kinkiensis* (*D.* sect. *Erythrovariae*; our Erythrovariae clade) and *D. pacifica* (Nakai) Tagawa (*D.* sect. *Variae*; our Variae clade) [59]. Using allozyme data Jiménez et al. [60] concluded that *D. corleyi*, an endemic of northern Spain, is of hybrid origin between *D. aemula* (*D.* sect. *Aemulae*) and *D. oreades* Fomin (*D.* sect. *Dryopteris*; not sampled in our study but would presumbly be in our Dryopteris clade). Our analyses based on plastid data and the resolution of *D. corleyi* as sister to *D. aemula* suggest that *D. aemula* is the maternal progenitor of *D. corleyi*. In addition, Sessa et al. [58] found evidence of extensive hybridization among the New World species of *Dryopteris* that has involved inter-continental long-distance dispersal as well as inter-clade hybridization. These examples of hybridization not only highlight the importance of reticulate evolution and thus the importance of nuclear data in understanding the evolutionary history of *Dryopteris*, but also strongly support the inclusion of these 13 lineages, including the small segregates, within *Dryopteris*, as opposed to breaking *Dryopteris* into many small genera.

### Major evolutionary lineages in *Dryopteris*

Within the newly defined *Dryopteris* (incl. *Acrorumohra*, *Dryopsis*, *Nothoperanema*, and Peranemataceae; Figure 1), the 100 species included in the current study are resolved into the following 13 well-supported major clades based on our four-locus plastid data set (Figure 1). Most of these major clades are also defined by morphological synapomorphies.

1. The Nothoperanema clade (ML BS: 98%; MP JK: 95%): This clade contains species of Peranemataceae sensu Ching [37], Wu [33,34], and Wu & Ching [35] and *Nothoperanema*. The potential morphological synapomorphies of this clade include the presence of non-glandular hairs and round and inferior indusia. The genus *Nothoperanema* is defined by the presence of thick, stout, and reddish brown setae.

2. The Aemulae clade (ML BS: 100%; MP JK: 100%): The Aemulae clade, or the Hawaiian glabra group [18], contains two species, *Dryopteris aemula* and *D. corleyi*, based on the current sampling. These two species are different enough morphologically to have been placed in different sections by Fraser-Jenkins [1]. Our resolution of *D. aemula* is consistent with those of Geiger & Ranker [18], Juslén et al. [57], and Sessa et al. [27]. This is not surprising because *D. corleyi*, an endemic of northern Spain, is thought to be of hybrid origin between *D. aemula* (*D.* sect. *Aemulae*) and *D. oreades* Fomin based on allozyme data ([60]; see below). All three species of *D.* sect. *Aemulae* sensu Fraser-Jenkins [1] are included in our analysis, but they are resolved as polyphyletic, with *D. chinensis* and *D. gymnophylla* grouping with *Acrorumohra* and *D. polita*. Based on Geiger & Ranker [18], Juslén et al. [57], and Sessa et al. [27] the Aemulae clade may also include *D. guanchica* Gibby & Jermy, *D. glabra* (Brackenr.) Kuntze, and *D. hawaiiensis* (Hillebrand) W. Robinson, but *D. guanchica* is an allotetraploid (see blow) and *D. hawaiiensis* possibly an allotriploid [61].

3. The Acrorumohra clade (ML BS: 86%; MP JK: 81%): This clade contains species of *Acrorumohra* and *Dryopteris chinensis*, *D. gymnophylla* (*D.* sect. *Aemulae*), and *D. polita* (*D.* sect. *Politae*). The morphological synapomorphy is the flat scales in comparison with the Dryopsis clade, the Erythrovariae clade, and the Variae clade. The gain of bullate scales is considered here as the morphological synapomorphy of the expanded *D.* subgen. *Erythrovariae* including the Acrorumohra clade, the Dryopsis clade, the Erythrovariae clade, and the Variae clade.

4. The Variae clade (ML BS: 95%; MP JK: 94%): The Variae clade contains species of *Dryopteris* sect. *Variae* Fraser-Jenk. It is characterized by having slightly bullate-based scales and stiffly coriaceous lamina and pinnules with caudate apices and pointed lobes [1,26].

5. The Erythrovariae clade (ML BS: 99%; MP JK: 98%): This clade contains species of *Dryopteris* sect. *Erythrovariae* plus *D. podophylla*. It is characterized by having more bullate scales and herbaceous lamina and pinnules with acute apices and rounded lobes [1,26].

6. The Dryopsis clade (ML BS: 76%; MP JK: 66%): This clade contains species of the former genus *Dryopsis*. The relatively moderate branch support may be the result of some missing sequence data for members of this clade. The potential major morphological synapomorphies are the rachis and costa grooves that are closed near their bases and the multi-cellular hairs (see above).

7. The Cinnamomeae clade (ML BS: 100%; MP JK: 100%): The Cinnamomeae clade contains two species of *Dryopteris* sect. *Cinnamomeae* and one species of *D.* sect. *Purpurascentes* in our current sampling. This clade is defined by having pinnules angled acroscopically and usually with narrower bases and having linear scales on stipe base [1].

8. The Crinales clade (ML BS: 100%; MP JK: 100%): This clade was named the Hawaiian exindusiate group by Palmer [62] and it contains five Hawaiian endemics, *Dryopteris crinalis* (Hook. & Arn.) C. Chr., *D. mauiensis* C. Chr., *D. sandwiciensis* (Hook. & Arn.) C. Chr., *D. tetrapinnata* W. H. Wagner & Hobdy, and *D. unidentata* (Hook. & Arn.) C. Chr. The potential morphological synapomorphy is the absence of indusia [18,62].

9. The Pallidae clade (ML BS: 97%; MP JK: 99%): This clade contains some species of *Dryopteris* sect. *Pallidae* sensu Fraser-Jenkins [1], e.g., *D. aitoniana* Pic. Serm., *D. odontoloma* (Bedd.) C. Chr., *D. pallida* (Bory) C. Chr. ex Maire & Petitm., and *D. mindshelkensis* N. Pavl. (synonym: *D. submontana* (Fraser-Jenk. & Jermy) Fraser-Jenk.), and additional species from other sections, e.g., *D. goldiana* (Hook.) A. Gray, *D. monticola* (Makino) (*D.* sect. *Dryopteris*), *D. oligodonta* (Desv.) Pic. Serm. (*D.* sect. *Marginatae*), and *D. tokyoensis* (Matsum. & Makino) C. Chr. (*D.* sect. *Pandae*), based our current sampling and Juslén et al. [57]. The inclusion of *D. odontoloma* in this clade needs further study. This clade is weakly supported as sister to the Crinales clade (Figure 1; ML BS: <50%; MP JK: 50%). In comparison with its sister, the Pallidae clade has indusia, but the Pallidae clade can be defined by having narrowly deltate-lanceolate leaves and stalked pinnules [1].

10. The Nephrocystis clade (ML BS: 100%; MP JK: 100%): This clade contains those species of *Dryopteris* sect. *Nephrocystis* sensu Fraser-Jenkins [1] with catadromous arrangement of leaf segments. It is characterized by having asymmetrical bases of basal pinnae with basiscopic pinnules much longer, and by having stipe-base scales that are lanceolate or ovate-lanceolate and brown [26].

11. The Dryopteris clade (ML BS: 99%; MP JK: 100%): This clade contains large portion of species of *Dryopteris* subgen. *Dryopteris* sensu Fraser-Jenkins [1] and Wu & Lu [26] and is the most species-rich clade of the genus. Most species of this clade have symmetrical pinnule bases (exceptions include *D. reflexosquamataD. rubrobrunnea*, etc.) and are mainly distributed in the Sino-Japanese and Sino-Himalayan regions.

12. The Lophodium clade (ML BS: 98%; MP JK: 100%): This clade contains species of *Dryopteris* sect. *Lophodium* Fraser-Jenk. and *D. remota*. The species of this clade share short-stalked pinnae, long-aristate ultimate segments, and minutely spinulose perispore sculpturing except *D. remota*[1].

13. The Fragrantes clade (ML BS: 100%; MP JK: 100%): This clade contains one of the two species of *Dryopteris* sect. *Fragrantes* (H. Itô) Seriz., *D. fragrans* (L.) Schott. Our work shows that *D. fragrans* is outside of *D.* subgen. *Dryopteris* where it was placed by Fraser-Jenkins [1] and Wu & Lu [26], a resolution consistent with that in Geiger & Ranker [18]. Most notably, our data agreed with Sessa et al. [27,58] in resolving *D. fragrans* as sister to the rest of *Dryopteris*, though our statistical support values were low (ML BS: <50%; MP JK: 60%).Our data clearly show that the Dryopsis clade is sister to the Erythrovariae clade; these two together are sister to a clade containing the Acrorumohra clade and the Variae clade; these four clades together are sister to a clade containing the Aemulae clade and the Nothoperanema clade; and these six clades are strongly supported as monophyletic (ML BS: 99%; MP JK: 94%). The relationships among the remaining seven clades are resolved in the ML tree (Figure 1) but with weak (<50%) branch support.

## Conclusions

The genera, *Acrophorus*, *Acrorumohra*, *Diacalpe*, *Dryopsis*, *Nothoperanema*, and *Peranema*, should all be merged into *Dryopteris*. Most species of these genera share a short rhizome and catadromic arrangement of frond segments, unlike the sister genus of *Dryopteris* s.l., *Arachniodes*.

The non-monophyly of the 19 out of the 21 supraspecific taxa in *Dryopteris* strongly suggests that the current taxonomy of this genus is in need of revision. However, our data do not necessarily falsify the monophyly of these 19 sections. The disagreement between previous taxonomy and molecular results in *Dryopteris* may be due partly to interspecific hybridization and polyplodization.

The 13 well-supported clades identified with our data represent 13 major evolutionary lineages in *Dryopteris* that are supported by morphological synapomophies and may deserve circumscription as supraspecific entities within *Dryopteris*.

## Materials and methods

### Taxon sampling

All four subgenera and 14 out of 16 sections of *Dryopteris* recognized by Fraser-Jenkins [1] and three additional sections (*D.* sect. *Caespitosae* S. G. Lu, *D.* sect. *Chrysocomae* S. G. Lu, *D.* sect. *Indusiatae* S. G. Lu) recognized by Lu [2,24,25] and partly by Wu & Lu [26], were represented by two to 12 species each. The only sections sampled that were represented by one species are *D.* sect. *Purpurascentes* Fraser-Jenk. and the monotypic *D.* sect. *Politae* Fraser-Jenk. In total, 78 accessions representing 77 species of *Dryopteris* s.s. were sampled, including all four subgenera and 19 sections in the current classifications of *Dryopteris* s.s. by Fraser-Jenkins [1], Lu [2,24,25], and Wu & Lu [26].

To assess the phylogenetic relationships between *Dryopteris* and *Acrophorus*, *Acrorumohra*, *Diacalpe*, *Dryopsis*, *Nothoperanema*, and *Peranema*, further included are eight accessions representing five (63%) out of eight species of *Acrophorus*, 10 accessions representing three (43%) out of seven species of *Acrorumohra*, seven accessions representing four (40%) out of 10 species of *Diacalpe*, six accessions representing five (31%) out of 16 species of *Dryopsis*, 11 accessions representing six (75%) out of eight species of *Nothoperanema*, and two accessions representing both species of the bitypic *Peranema*. Type species of all these six genera, *Acrophorus*, *Acrorumohra*, *Diacalpe*, *Dryopsis*, *Nothoperanema*, and *Peranema*, are included. In total, 122 accessions representing 100 species of the expanded *Dryopteris* (incl. *Acrophorus*, *Acrorumohra*, *Diacalpe*, *Dryopsis*, *Nothoperanema*, and *Peranema*) are included in this study.

One species each of *Arachniodes* Blume, *Leptorumohra* H. Itô, and *Lithostegia* Ching of Dryopteridaceae are used as outgroups based on Liu et al. [21] where *ArachniodesLeptorumohraLithostegia*, and *Phanerophlebiopsis* Ching together were resolved as sister to a clade consisting of *AcrophorusAcrorumohraDiacalpeDryopsisDryopterisNothoperanema*, and *Peranema*. All sequences used in this study together with their GenBank accession numbers and/or voucher information are listed in Appendix II.

### DNA sequencing

Total genomic DNA was extracted from silica-gel dried material or sometimes from herbarium specimens using Plant Genomic DNA Kits (TIANGEN BioTech., Beijing, China) and DNeasy Plant Mini Kits (Qiagen, Germany). The PCR protocols followed Zhang et al. [63] and Ebihara et al. [64]. DNA sequence data were obtained for four plastid regions, *rbcL* gene, *rps4-trnS* spacer, *trnL* intron, and *trnL-F* spacer. The *rbcL* gene was amplified with primers F1 (5’-ATGTCACCACAAACAGAAACTAAAGC; Fay et al. [65]) and 1379R (5’-TCACAAGCAGCAGCTAGTTCAGGACTC; originally designed by G. Zurawski and modified by Wolf et al. [66]). The primers for amplifying *rps4-trnS* intergenic spacer were derived from Souza-Chies et al. (5’-TACCGAGGGTTCGAATC; [67]) and Li & Lu (5’-ATGAATT(A/G)TTAGTTGTTGAG; [19]). The *trnL* intron and *trnL-F* intergenic spacer were amplified using the primers fern 1 (5’-GGCAGCCCCCARATTCAGGGRAACC; [68]) and the universal primer f (5’-ATTTGAACTGGTGACACGAG) of Taberlet et al. [69]. Amplified fragments were purified with TIANquick Mini Purification Kits (TIANGEN) and ExoSAP-IT (USB, CA, USA). Purified PCR products were sequenced by Invitrogen^TM^ (Shanghai, China) and BigDye® Terminator v3.1 Cycle Sequencing Kit (Applied Biosystems, CA, USA).

Additional sequences were obtained from Genbank and had originally been generated by Geiger & Ranker [18], Li & Lu [19,20], Ebihara et al. [64], de Groot et al. [70], Juslén et al. [57], and Sessa et al. [27,58]. In total, 114, 92, 68, and 111 sequences of *rbcLrps4-trnStrnL*, and *trnL-F*, respectively, were included in our analyses. Some 151 DNA sequences are newly generated for this study (GenBank JX535813-JX535961).

### Molecular phylogenetics

The alignment of the *rbcL* data was manually obtained using Microsoft Wordpad. Preliminary alignments of *rps4-trnS* and *trnL-F* (*trnL* intron + *trnL-F* spacer) data were obtained using the default alignment parameters in Clustal X [71] followed by manual adjustments. Gap characters were coded as missing data.

Equally weighted maximum parsimony (MP) tree searches were conducted for each data matrix using 1000 tree-bisection-reconnection (TBR) searches in PAUP* ver. 4.0b10 [72] with MAXTREES set to increase without limit. Parsimony jackknife (JK) analyses [73] were conducted using PAUP* with the removal probability set to approximately 37%, and “jac” resampling emulated. One thousand replicates were performed with 10 TBR searches per replicate and a maximum of 100 trees held per TBR search. In addition to the analyses of the four individual regions, MP and ML analyses of the combined *trnL* intron and *trnL-F* spacer were also conducted since these two linked regions are sometimes viewed as one locus.

MrModeltest 2.3 [74], a modified version of Modeltest 3.6 [75], was used to select the best fit likelihood model for maximum likelihood (ML; [76]) analyses. The Akaike Information Criterion [77] was used to select among models instead of the hierarchical likelihood ratio test, following Pol [78] and Posada and Buckley [79]. The models selected were GTR + G (*trnL-F* spacer), GTR + I (*trnL* intron), GTR + I + G (*trnL* intron &*trnL-F* spacer and the simultaneous analysis), HKY + G (*rps4-trnS* spacer), and SYM + I + G (*rbcL* gene). The selected models and parameters estimated (Table 2) were then used for tree searches from the respective data partitions. One hundred jackknife replicates were performed with one TBR search per replicate and a maximum of 100 trees held per TBR search. 

**Table 2:** **Best-fitting models and parameter values for separate (*rbcL*,*rps4-trnS*,*trnL, trnL-F*, and*trnL*&*trnL-F*) and simultaneous plastid datasets in this study**

**Region**	**AIC selected model**	**Base frequencies**	**Substitution model (rate matrix)**	**I**	**G**
		A	A–C	A–G	A–T
*rbcL* gene	SYM+I+G	–	1.3128	7.2418	1.3793
*rps4-trnS* spacer	HKY+G	0.3065	–	–	–
*trnL* intron	GTR+I	0.3176	1.6096	5.9476	0.4952
*trnL-F* spacer	GTR+G	0.3349	0.6845	5.1845	0.1986
*trnL* intron &*trnL-F* spacer	GTR+I+G	0.3237	1.1111	5.5874	0.3645
simultaneous	GTR+I+G	0.2970	1.0836	5.9681	0.7802

“G” = gamma distribution shape parameter [82]. “GTR” = general-time-reversible model [83]. “HKY” = Hasegawa-Kishino-Yano model [84]. “I” = proportion of invariable sites. “SYM” = symmetrical model [85]. “Ti/Tv” = transition/transversion ratio.

The simultaneous ML analyses of nucleotide characters and ML bootstrapping (BS) were performed using RAxML-HPC2 on TG ver. 7.2.8 ([80,81]; available at http://www.phylo.org/) with 1000 rapid bootstrap analyses followed by a search for the best-scoring tree in a single run.

## Appendix I

### Monophylies of supraspecific taxa in *Dryopteris*

Our results show that none of the four subgenera, *D.* subgen. *DryopterisD.* subgen. *ErythrovariaeD.* subgen. *Nephrocystis*, and *D.* subgen. *Pycnopteris* (T. Moore) Ching, are monophyletic. Sessa et al. [27] also rejected monophyly of these subgenera, except for *D.* subgen *Pycnopteris*, for which they had insufficient sampling to test monophyly. In the current study, most of the members of *D.* subgen. *Dryopteris* are resolved in the Dryopteris clade, while others are placed in other major clades except the Acrorumohra*,* Dryopsis*,* Erythrovariae*,* Nothoperanema*,* and Variae clades. Members of *D.* subgen. *Erythrovariae* sensu Fraser-Jenkins [1] are resolved in the Acrorumohra*,* Erythrovariae, and Variae clades, but these clades are paraphyletic in relation to the Dryopsis clade as well as *D. chinensis* (Baker) Koidz. and *D. gymnophylla* (Baker) C. Chr. (members of *D.* sect. *Aemulae* Fraser-Jenk.), and *D. podophylla* (Hook.) Kuntze (a member of *D.* subgen. *Pycnopteris)*. Members of *D.* subgen. *Erythrovariae* sensu Wu & Lu [26] are resolved in the Erythrovariae and Variae clades. The non-monophyly of *D.* subgen. *Pycnopteris* is consistent with Li & Lu’s [19,20] finding based on *rps4-trnS* data. Of our three representatives of this subgenus, two fell in the Dryopteris clade (*D. bodinieri* (Christ) C. Chr. and *D. sieboldii* (Van Houtte ex Melt.) Kuntze), and one in the Erythrovariae clade (*D. podophylla*). Our sole sequence of *D. podophylla* was derived from Li & Lu [19]. *D.* subgen. *Nephrocystis* is not monophyletic because *Acrorumohra diffracta* Baker (= *D. diffracta* (Baker) C. Chr.), *A. hasseltii* Blume (=*D. hasseltii* (Blume) C. Chr.), *A. subreflexipinna* (Ogata) Ching (= *D. subreflexipinna* Ogata), and *D. futura* A. R. Sm., a member of *D.* sect. *Purpurascentes*, are resolved in the Acrorumohra clade and the Cinnamomeae clade. Wu & Lu [26] did not recognize *D.* subgen. *Nephrocystis*.

Our results also demonstrated that 15 out of the 17 sections currently recognized [1,2,24-26], for which two or more species are sampled, are not monophyletic (Figure 1). Only two sections, *D.* sect. *Cinnamomeae* Fraser-Jenk. and *D.* sect. *Variae* Fraser-Jenk., are resolved as monophyletic with our current sampling. This is at odds with Sessa et al. [27], which tested the monophly of eleven sections, including *D.* sect. *Cinnamomeae* and *D.* sect. *Variae*, and rejected the monophly of all. Although these two sections are found to be monophyletic in the current study, the sampling for both was larger in Sessa et al. [27], and they are thus not likely to be monophyletic either. The 15 polyphyletic sections discovered in our study are:

*Dryopteris* sect. *Aemulae* Fraser-Jenk.: Represented by *D. aemula* (Aiton) Kuntze (Aemulae clade), *D. chinensis*, and *D. gymnophylla* (Acrorumohra clade).

*Dryopteris* sect. *Caespitosae* S. G. Lu: Represented by *D. alpestris* Tagawa (Dryopteris clade) and *D. fragrans* (Fragrantes clade).

*Dryopteris* sect. *Chrysocomae* S. G. Lu: Represented by *D. chrysocoma* (Christ) C. Chr. and *D. himachalensis* Fraser-Jenk. (Dryopteris clade).

*Dryopteris* sect. *Dryopteris*: This section sensu Fraser-Jenkins [1] is represented by *D. alpestrisD. filix-mas* (L.) Schott, and *D. sichotensis* V. Komarov (Dryopteris clade).

*Dryopteris* sect. *Fibrillosae* Ching: Represented by *D. affinis* (Lowe) Fraser-Jenk. subsp. *borreri* Fraser-Jenk., *D. polylepis* (Franchet & P. A. L. Saval.) C. Chr., and *D. rosthornii* (Diels) C. Chr. (Dryopteris clade).

*Dryopteris* sect. *Erythrovariae*: This section sensu Fraser-Jenkins [1] is represented by *D. caudipinna* Nakai, *D. championii* (Benth.) C. Chr., *D. cordipinna* Ching & Shing, *D. decipiens* (Hook.) Kuntze, *D. erythrosora* (D. Eaton) Kuntze, *D. gymnosora* (Makino) C. Chr., *D. indusiata* Makino & Yamam. (= *D. tenuicula* C. Matthew & Christ following Fraser-Jenkins [1]), *D. kinkiensis* Koidz. ex Tagawa, *D. nipponensis* Koidz. (= *D. cystolepidota* (Miq.) Makino following Fraser-Jenkins [1]), *D. ryo-itoana* Kurata, and *D. simasakii* (H. Itô) Kurata and all are in the Erythrovariae clade. If *D. podophylla* (Hook.) Kuntze, a member of *D.* subgen. *Pycnopteris*, is included, this section sensu Fraser-Jenkins [1] becomes monophyletic.

*Dryopteris* sect. *Hirtipedes* Fraser-Jenk.: Represented by *D. atrata* (Wall) Ching, *D. commixta* Tagawa, *D. conjugata* Ching, *D. cycadina* (Franchet & P. A. L. Savat.) C. Chr., *D. dickinsii* (Franchet & P. A. L. Savat.) C. Chr., *D. handeliana* C. Chr., *D. hangchowensis* Ching, *D. lunanensis* (Christ) C. Chr., *D. scottii* (Bedd.) Ching, *D. stenolepis* (Baker) C. Chr. and *D. tsutsuiana* Kurata, all of which are in the Dryopteris clade. This section becomes monophyletic if *D. rosthornii* (*D.* sect. *Fibrillosae*) and *D. uniformis* (*D.* sect. *Pallidae*) are included.

*Dryopteris* sect. *Indusiatae* S. G. Lu: Represented by *D. gymnosora* (Makino) C. Chr. and *D. indusiata* Makino & Yamam. (Erythrovariae clade).

*Dryopteris* sect. *Lophodium* (Newman) C. Chr. ex H. Itô: Represented by *D. amurensis* Christ, *D. expansa* (C. Presl) Fraser-Jenk. & Jermy, *D. intermedia* (Muhlenb. ex Willd.) A. Gray, and *D. dilatata* (Hoffm.) A. Gray (Lophodium clade). These four species are paraphyletic in relation to *D. remota* (A. Braun ex Doell) Druce, the type of *D.* sect. *Remotae*. The close relationship between *D. remota* and species of *D.* sect. *Lophodium* based on our plastid data shows that *D. remota*, a triploid, is possibly originated through hybridization with one of the species in *D.* sect. *Lophodium* being the maternal donor.

*Dryopteris* sect. *Marginatae* Fraser-Jenk.: Represented by *D. aquilinoides* (Desv.) C. Chr. and *D. shiroumensis* Kurata & Nakaike (Dryopteris clade). They are resolved as paraphyletic in relation to the rest of the Dryopteris clade.

*Dryopteris* sect. *Nephrocystis*: Represented by *Acrorumohra diffractaA. hasseltiiA. subreflexipinnaDryopteris hayatae* Tagawa (subsumed in *D. subexaltata* (Christ) C. Chr. by Fraser-Jenkins [1]), *D. melanocarpa* Hayata (subsumed in *D. platypus* (Kunze) Kuntze by Fraser-Jenkins [1]), *D. maximowicziana* (Miq.) C. Chr. (not recognized by Fraser-Jenkins [1]), *D. sabae* (Franchet & P. A. L. Savat.) C. Chr., *D. sparsa* (Buch.-Ham. ex D. Don) Kuntze, and *D. yakusilvicola* Sa. Kurata (subsumed in *D. cacaiana* Tagawa by Fraser-Jenkins [1]). The first three species are resolved in the Acrorumohra clade while the rest are in the Nephrocystis clade.

*Dryopteris* sect. *Pallidae* Fraser-Jenk.: Represented by *D. juxtaposita* Christ, *D. lacera* (Thunb.) Kuntze, *D. sublacera* Christ, *D. uniformis* (Makino) Makino (Dryopteris clade), *D. odontoloma* (Bedd.) C. Chr., and *D. pallida* (Bory) C. Chr. ex Maire & Petitm. (Pallidae clade).

*Dryopteris* sect. *Pandae* Fraser-Jenk.: Represented by *D. himachalensis* Fraser-Jenk. (Acrorumohra clade) and *D. tokyoensis* (Matsum. & Makino) C. Chr. (Pallidae clade).

*Dryopteris* sect. *Remotae* Fraser-Jenk.: Represented by *D. corleyi* Fraser-Jenk. (Aemulae clade) and *D. remota* (Lophodium clade).

*Dryopteris* sect. *Splendentes* Fraser-Jenk.: Represented by *D. reflexosquamata* Hayata and *D. rubrobrunnea* W. M. Chu (Dryopteris clade). These two are paraphyletic in relation to three members of *D.* sect. *Pallidae* and two member of *D.* subgen. *Pycnopteris*.

## Appendix II

### List of taxa sampled with information related to taxonomy, voucher information and GenBank accession numbers

*Acrophorus emeiensis* Ching: *rbcL* zl1474, *trnL* JX535916, *trnL-F* JX535867, *rps4-trnS* JX535815. *Acrophorus exstipellatus* Ching & S. H. Wu: *rbcL* JX535857, *trnL* JX535914, *trnL-F* JX535865, *rps4-trnS* JX535813. *Acrophorus macrocarpus* Ching & S. H. Wu: *rbcL* DQ054522 (“*Acrophorus emeiensis* Ching”). *Acrophorus nodosus* C. Presl: *rbcL* AB575065, *trnL* JX535915, *trnL-F* JX535866, *rps4-trnS* JX535814. *Acrophorus paleolatus* Pic. Serm. (“*Acrophorus stipellatus* T. Moore”): *rbcL* DQ054510 DQ508756 EF463106, *trnL-F* DQ514500 EF540696 DQ480130.

*Acrorumohra diffracta* (Baker) H. Itô: *rbcL* DQ508758 EF463108, *trnL-F* EU797681 EU797682 EU797683, *rps4-trnS* EU797685 EU797686 EU797687. *Acrorumohra hasseltii* (Blume) Ching: *rbcL* AB575136 DQ054519 DQ508757 EF463107, *trnL-F* DQ514479 EU797677 EU797679 EU797680, *rps4-trnS* DQ191888 EU797691 EU797692 EU797693. *Acrorumohra subreflexipinna* (Ogata) H. Itô: *trnL -F* EU797675 EU797676 EU797678, *rps4-trnS* EU797688 EU797689 EU797690.

*Arachniodes aristata* (G. Forst.) Tindale: *rbcL* AY268851, *trnL-F* AY268782. *Arachniodes assamica* (Kuhn) Ohwi: *rps4-trnS* DQ191891.

*Diacalpe annamensis* Tagawa: *rbcL* EF463125, *trnL-F* DQ480132 EF540698. *Diacalpe aspidioides* Blume: *rbcL* DQ054523 EF463126, *trnL-F* DQ514490. *Diacalpe chinensis* Ching & S. H. Wu: *rbcL* JX535864, *trnL* JX535956, *trnL-F* JX535908, *rps4-trnS* JX535854. *Diacalpe christensenae* Ching: *rbcL* DQ054518 EF540699, *trnL-F* DQ480131 EF540699, *rps4-trnS* DQ480131 EF540699.

*Dryopsis apiciflora* (Wall. ex Mett.) Holttum & P.J. Edwards: *rbcL* DQ054521, *trnL* JX535957, *trnL-F* JX535909. *Dryopsis clarkei* (Baker) Holttum & P.J. Edwards: *trnL* JX535958, *trnL-F* JX535910, *rps4-trnS* JX535855. *Dryopsis heterolaena* (C. Chr.) Holttum & P.J. Edwards: *rbcL* DQ508770, *trnL-F* DQ514492. *Dryopsis mariformis* (Rosenst.) Holttum & P.J. Edwards: *rbcL* DQ054520 EF460683, *trnL* JX535959, *trnL-F* JX535911. *Dryopsis* sp.: *rbcL* DQ054525.

*Dryopteris aemula* (Aiton) Kuntze: *rbcL* AY268881, *trnL-F* AY268816, *rps4-trnS* JN189189. *Dryopteris affinis* (Lowe) Fraser-Jenk. subsp. *borreri* Fraser-Jenk.: *rbcL* AY268849, *trnL-F* AY268776, *rps4-trnS* JN189190. *Dryopteris alpestris* Tagawa: *rbcL* JX535858, *trnL* JX535917, *trnL-F* JX535868, *rps4-trnS* JXH11103. *Dryopteris amurensis* Christ: *rbcL* AB575112, *trnL* JX535918, *trnL-F* JX535869, *rps4-trnS* JX535816. *Dryopteris aquilinoides* (Desv.) C. Chr.: *rbcL* AY268868, *trnL-F* AY268803, *rps4-trnS* JN189211. *Dryopteris atrata* (Wall) Ching: *rbcL* AB575115, *trnL* JX535919, *trnL-F* JX535870, *rps4-trnS* JX535817. *Dryopteris bissetiana* (Baker) C. Chr.: *rbcL* AY268862, *trnL-F* AY268796, *rps4-trnS* DQ191829. *Dryopteris bodinieri* (Christ) C. Chr.: *rbcL* DQ508772, *trnL-F* DQ514494, *rps4-trnS* DQ191830. *Dryopteris caudipinna* Nakai: *rbcL* AB575117, *trnL* JX535920, *trnL-F* JX535871, *rps4-trnS* JX535818. *Dryopteris championii* (Benth.) C. Chr.: *rbcL* AY268863, *trnL-F* AY268797, *rps4-trnS* DQ151856. *Dryopteris chinensis* (Baker) Koidz.: *rbcL* JX535859, *trnL* JX535921, *trnL-F* JX535872, *rps4-trnS* JX535819. *Dryopteris chrysocoma* (Christ) C. Chr.: *rbcL* DQ508773, *trnL-F* DQ514495, *rps4-trnS* DQ191832. *Dryopteris cinnamomea* (Cav.) C. Chr.: *rbcL* JN189528, *trnL-F* FR731991, *rps4-trnS* JN189202. *Dryopteris commixta* Tagawa: *rbcL* AB575120, *trnL* JX535922, *trnL-F* JX535873, *rps4-trnS* JX535820. *Dryopteris corleyi* Fraser-Jenk.: *rbcL* AY268873, *trnL-F* AY268808. *Dryopteris crinalis* (Hook. &Arn.) C. Chr.: AY268835, *trnL-F* AY268774. *Dryopteris cycadina* (Franchet & P. A. L. Savat.) C. Chr.: *rbcL* EF463127, *trnL-F* AY278400, *rps4-trnS* DQ191835. *Dryopteris decipiens* (Hook.) Kuntze var. *decipiens*: *rbcL* AB575123, *trnL* JX535923, *trnL-F* JX535874, *rps4-trnS* JX535821. *Dryopteris dickinsii* (Franchet & P. A. L. Savat.) C. Chr.: *rbcL* AB575125, *trnL* JX535924, *trnL-F* JX535875, *rps4-trnS* JX535822. *Dryopteris dilatata* (Hoffm.) A. Gray: *rbcL* AY268848, *trnL-F* AY268779, *rps4-trnS* JN189248. *Dryopteris erythrosora* (D. Eaton) Kuntze: *rbcL* DQ508774, *trnL-F* DQ514496, *rps4-trnS* JN189255. *Dryopteris expansa* (C. Presl) Fraser-Jenk. & Jermy: *rbcL* AY268844, *trnL-F* AY268775, *rps4-trnS* JN189180. *Dryopteris filix-mas* (L.) Schott: *rbcL* AY268845, *trnL-F* AY268776, *rps4-trnS* JN189181. *Dryopteris fragrans* (L.) Schott: *rbcL* AB575129, AY268865, *trnL-F* FR731981 AY268800, *rps4-trnS* JN189185. *Dryopteris futura* A. R. Sm.: *rbcL* JN189534, *trnL-F* JN189103, *rps4-trnS* JN189208. *Dryopteris gymnophylla* (Baker) C. Chr.: *rbcL* JX535860, *trnL* JX535925, *trnL-F* JX535876, *rps4-trnS* JX535823. *Dryopteris gymnosora* (Makino) C. Chr.: *rbcL* AB575132, *trnL* JX535926, *trnL-F* JX535877, *rps4-trnS* JX535824. *Dryopteris hadanoi* Kurata: *rbcL* AB575133, *trnL* JX535927, *trnL-F* JX535878, *rps4-trnS* JX535825. *Dryopteris handeliana* C. Chr.: *rbcL* AB575134, *trnL* JX535928, *trnL-F* JX535879, *rps4-trnS* JX535826. *Dryopteris hangchowensis* Ching: *rbcL* AB575135, *trnL* JX535929, *trnL-F* JX535880, *rps4-trnS* JX535827. *Dryopteris hayatae* Tagawa: *rbcL* AB575137, *trnL* JX535930, *trnL-F* JX535881, *rps4-trnS* JX535828. *Dryopteris himachalensis* Fraser-Jenk.: *rps4-trnS* DQ191845. *Dryopteris indusiata* Makino & Yamam.: *rbcL* AB575140, *trnL* JX535931, *trnL-F* JX535882, *rps4-trnS* JX535829. *Dryopteris intermedia* (Muhlenb. ex Willd.) A. Gray subsp. *maderensis* (J. Milde ex Alston) Fraser-Jenkins: *rbcL* AB575143, *trnL-F* FR731985. *Dryopteris juxtaposita* Christ: *rbcL* AY268875, *trnL-F* AY268810, *rps4-trnS* DQ191848. *Dryopteris kinkiensis* Koidz. ex Tagawa: *rbcL* AB575144, *trnL* JX535932, *trnL-F* JX535883, *rps4-trnS* JX535830. *Dryopteris lacera* (Thunb.) Kuntze: *rbcL* AB575148, *trnL* JX535933, *trnL-F* JX535884, *rps4-trnS* JX535831. *Dryopteris laeta* (Kom.) C. Chr.: *rbcL* AB575149, *trnL* JX535934, *trnL-F* JX535885, *rps4-trnS* JX535832. *Dryopteris lunanensis* (Christ) C. Chr.: *rbcL* AB575150, *trnL* JX535935, *trnL-F* JX535886, *rps4-trnS* JX535833. *Dryopteris mauiensis* C. Chr.: *rbcL* AY268833, *trnL-F* AY268770. *Dryopteris maximowicziana*: *rbcL* AB575151, *trnL* JX535936, *trnL-F* JX535887, *rps4-trnS* JX535834. *Dryopteris melanocarpa* Hayata: *rbcL* AB575153, *trnL* JX535937, *trnL-F* JX535888, *rps4-trnS* JX535835. *Dryopteris monticola* (Makino) C. Chr.: *rbcL* AB575154, *trnL* JX535938, *trnL-F* JX535889, *rps4-trnS* JX535836. *Dryopteris nipponensis* Koidz.:AB575156, *trnL* JX535939, *trnL-F* JX535890, *rps4-trnS* JX535837. *Dryopteris odontoloma* (Bedd.) C. Chr.: *rbcL* AY268872, *trnL-F* AY268807, *rps4-trnS* DQ191859. *Dryopteris pacifica* (Nakai) Tagawa: *rbcL* AB575157, *trnL* JX535940, *trnL-F* JX535891, *rps4-trnS* JX535838. *Dryopteris pallida* (Bory) C. Chr. ex Maire & Petitm.: *rbcL* AY268874, *trnL-F* AY268809, *rps4-trnS* JN189266. *Dryopteris patula* (Sw.) L. Underw.: *rbcL* JN189500, *trnL-F* AY268823, *rps4-trnS* JN189176. *Dryopteris podophylla* (Hook.) Kuntze: *rps4-trnS* DQ191864. *Dryopteris polita* Rosenst.: *rbcL* AB575158, *trnL-F* EU797684, *rps4-trnS* EU797694. *Dryopteris polylepis* (Franchet & P. A. L. Saval.) C. Chr.: *rbcL* AY268864, *trnL-F* AY268798, *rps4-trnS* JN189263. *Dryopteris reflexosquamata* Hayata: *rbcL* JN189604, *trnL-F* JN189171, *rps4-trnS* DQ191870. *Dryopteris remota* (A. Braun ex Doell) Druce: *rbcL* AY268858, *trnL-F* AY268792, *rps4-trnS* JN189204. *Dryopteris rosthornii* (Diels) C. Chr.: *rbcL* JX535861, *trnL* JX535941, *trnL-F* JX535892, *rps4-trnS* JX535839. *Dryopteris rubrobrunnea* W. M. Chu: *rbcL* JX535862, *trnL* JX535942, *trnL-F* JX535893, *rps4-trnS* JX535840. *Dryopteris ryo-itoana* Kurata: *rbcL* AB575161, *trnL* JX535943, *trnL-F* JX535894, *rps4-trnS* JX535841. *Dryopteris sabae* (Franchet & P. A. L. Savat.) C. Chr.: *rbcL* AB575162, *trnL* JX535944, *trnL-F* JX535895, *rps4-trnS* JX535842. *Dryopteris sacrosancta* Koidz.: *rbcL* AB575163, *trnL* JX535945, *trnL-F* JX535896, *rps4-trnS* JX535843. *Dryopteris sandwiciensis* (Hook. & Arn.) C. Chr.: *rbcL* AY268827, *trnL-F* AY268762. *Dryopteris saxifraga* H. Itô: *rbcL* AB575164, *trnL* JX535946, *trnL-F* JX535897, *rps4-trnS* JX535844. *Dryopteris scottii* (Bedd.) Ching: *rbcL* JX535863, *trnL-F* JX535898, *rps4-trnS* DQ191872. *Dryopteris shiroumensis* Kurata & Nakaike: *rbcL* AB575168, *trnL* JX535947, *trnL-F* JX535899, *rps4-trnS* JX535845. *Dryopteris sichotensis* V. Komarov: *rbcL* AY268869, *trnL-F* AY268804. *Dryopteris sieboldii* (Van Houtte ex Melt.) Kuntze: *rbcL* AB575169, *trnL* JX535948, *trnL-F* JX535900, *rps4-trnS* JX535846. *Dryopteris simasakii* (H. Itô) Kurata var. *simasakii*: *rbcL* AB575170, *trnL* JX535949, *trnL-F* JX535901, *rps4-trnS* JX535847. *Dryopteris sordidipes* Tagawa: *rbcL* AB575172, *trnL* JX535950, *trnL-F* JX535902, *rps4-trnS* JX535848. *Dryopteris sparsa* (Buch.-Ham. ex D. Don) Kuntze: *rbcL* AB575173, *trnL* JX535951, *trnL-F* JX535903, *rps4-trnS* JX535849. *Dryopteris stenolepis* (Baker) C. Chr.: *rbcL* AY268889, *trnL-F* AY268824, *rps4-trnS* DQ191877. *Dryopteris sublacera* Christ: *rbcL* DQ508778, *trnL-F* DQ514501, *rps4-trnS* DQ191878. *Dryopteris tetrapinnata* W. H. Wagner & Hobdy: *rbcL* AY268838, *trnL-F* AY268772. *Dryopteris tokyoensis* (Matsum. & Makino) C. Chr.: *rbcL* AY268861, *trnL-F* AY268795, *rps4-trnS* JN189251. *Dryopteris tsutsuiana* Kurata: *rbcL* AB575176, *trnL* JX535952, *trnL-F* JX535904, *rps4-trnS* JX535850. *Dryopteris unidentata* (Hook. & Arn.) C. Chr. var. *unidentata*: *rbcL* AY268825, *trnL-F* AY268766. *Dryopteris uniformis* (Makino) Makino: *rbcL* AB575177, *trnL* JX535953, *trnL-F* JX535905, *rps4-trnS* JX535851. *Dryopteris varia* (L.) Kuntze: *rbcL* AB575178, *trnL* JX535954, *trnL-F* JX535906, *rps4-trnS* JX535852. *Dryopteris xanthomelas* (Christ) C. Chr.: *rbcL* AY587118, *trnL-F* DQ150394, *rps4-trnS* DQ151857. *Dryopteris yakusilvicola* Sa. Kurata: *rbcL* AB575180, *trnL* JX535955, *trnL-F* JX535907, *rps4-trnS* JX535853.

*Leptorumohra quadripinnata* (Hayata) H. Itô: *rbcL* DQ508781, *trnL-F* DQ514505, *rps4-trnS* EF540707.

*Lithostegia foeniculacea* (Hook.) Ching: *rbcL* DQ508782, *trnL-F* DQ514506, *rps4-trnS* EF540717.

*Nothoperanema diacalpioides* Ching: *rbcL* DQ054511. *Nothoperanema hendersonii* (Bedd.) Ching: *rbcL* AB575138 DQ508783 EF463135 JN189547, *trnL-F* DQ514507 JN189116, *rps4-trnS* DQ191885 JN189221. *Nothoperanema rubiginosum* (Brack.) A. R. Sm. & D. R. Palmer: *rbcL* AY268836 DQ054511 (“*Nothoperanema hendersonii* (Bedd.) Ching”) EF463182 (“*Nothoperanema squamisetum* (Hook.) Ching”), *trnL-F* AY268771. *Nothoperanema shikokianum* (Makino) Ching: *rbcL* AB575167 DQ054509 EF463136, *trnL* JX535960, *trnL-F* JX535912, *rps4-trnS* DQ191886 JX535856. *Nothoperanema squamisetum* (Hook.) Ching: *rbcL* DQ054512, *trnL* JX535961, *trnL-F* JX535913, *rps4-trnS* DQ191887.

*Peranema cyatheoides* D. Don: *rbcL* DQ054513. *Peranema luzonicum* Copel.: *rbcL* DQ508784 (“*Peranema cyatheoides* D. Don”), *trnL-F* DQ514509 (“*Peranema cyatheoides* D. Don”).

## Competing interests

The author(s) declare that they have no competing interests.

## Authors’ contributions

LBZ desgined the study, conducted data analyses, and wrote the manuscript, LZ, EBS, and AE carried out the lab work, LZ, SYD, EBS, XFG, and AE collected and identified portion of the material. All authors contributed to the manuscript revision. All authors read and approved the final manuscript.
